# Effect of Solvents, Their Mixture and Thermal Annealing on the Performance of Solution Processed Polymer Light-Emitting Diodes

**DOI:** 10.3390/ma6051994

**Published:** 2013-05-15

**Authors:** Mohammad Hashem Rezvani, Farid Farajollahi, Alireza Nikfarjam, Parisa Bakhtiarpour, Erfan Saydanzad

**Affiliations:** 1 Nanoelectronics Research Group, Academic Center for Education, Culture and Research (ACECR), Khajeh Nasir Toosi University of Technology, Tehran 15569-14846, Iran; E-Mail: erfan_saydanzad@yahoo.com; 2 Institute for Experimental Physics, Ulm University, Ulm D-89081, Germany; E-Mails: farid.farajollahi@uni-ulm.de (F.F.); parisa.bakhtiarpour@uni-ulm.de (P.B.); 3 Faculty of New Sciences and Technologies, University of Tehran, Tehran 14395-1374, Iran; E-Mail: a.nikfarjam@ut.ac.ir

**Keywords:** aromatic solvent, post-thermal annealing, pre-thermal annealing, polymer light emitting diode, interface, semiconductor polymer, solvent mixture

## Abstract

In this study, we first investigated changes seen in electrical and optical properties of a polymer light-emitting diode due to using different kinds of solvents and their mixture. Two-layer light emitting diodes with organic small molecules doped in a PVK polymer host were fabricated using (i) non-aromatic solvent chloroform with a high evaporation rate; (ii) aromatic solvent chlorobenzene with a low evaporation rate, and (iii) their mixture with different relative ratios. The effect of nano-scale layer thickness, surface roughness and internal nano-morphology on threshold voltage and the amount of electric current, the luminance and efficiency of a device were assessed. Results indicated the importance of majority charge carriers’ type in the selection of solvent and tuning its properties. Then, the effect of thermal annealing on electrical and optical properties of polymer light emitting diodes was investigated. During the device fabrication, pre-annealing in 80 and/or 120 °C and post-annealing in 120 °C were performed. The nano-scale effect of annealing on polymer-metal interface and electric current injection was described thoroughly. A comparison between threshold voltage, luminance and electric current efficiency of luminescence for different annealing processes was undertaken, so that the best electric current efficiency of luminescence achieved at 120 °C pre-annealing accompanied with 120 °C post-annealing.

## 1. Introduction

Semiconductor polymers and organic materials have been increasingly used in electrical and optical devices [1,2,3,4,5]. Capability of electrical charge transfer and doping with different dyes makes them excellent candidates for organic light emitting diodes (OLED) [6,7,8,9,10]. Due to weak molecular interactions in organic materials and random orientation of polymer chains to each other, the performance of these devices has significant dependence on the fabrication process. 

Exploiting spin coating of organic material solutions makes the fabrication process of electronic and photonic products less complicated, and facilitates using their high application-oriented potential at a low cost [11,12]. 

The properties of a solvent have a critical role in the performance of devices fabricated through the spin coating method [11,13,14,15]. Tuning these properties could be an effective route for changing the behavior and improving the electrical and optical performance of the devices. 

In polymer light emitting diodes (PLED), different factors, e.g., type of solvent, spinning speed and substrate material affect the performance of the device [16,17,18]. Applying electric field, using ultra-violet radiation and thermal annealing are some of the routes can be exploited to change and improve the polymer morphology [19,20,21,22,23]. The nano-morphology of polymer thin films is one of the most important sources determining the polymeric device performance [17,24,25,26]. 

Thermal treatment of PLEDs in different steps of their fabrication process may increase their efficiency and improve their performance. Polymer has a glass transition temperature in which its phase changes from solid to liquid gradually. Thus, choosing appropriate temperature, the quality of polymer thin film interfaces can be changed in nano-scale without significant damage to overall diode structure.

**Scheme 1:**
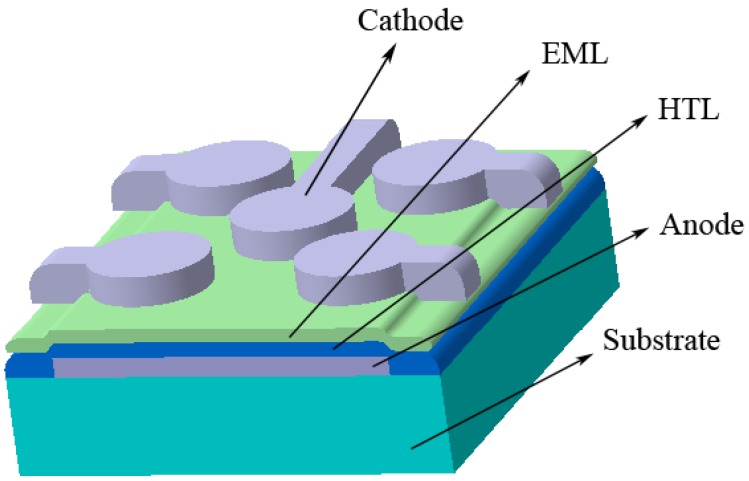
Schematic diagram of fabricated polymer light emitting diodes by solution processing.

In this paper, the fabrication of PLEDs is reported and an investigation is undertaken on the properties of PLEDs, with a focus on tuning and improvement of device properties without changing its structure (Scheme 1). First, we study the effect of solvent types and their mixture on the electrical and optical properties of device, and discuss the mechanism underlies this effect. Then, the effect of thermal annealing in different steps of the fabrication process on the electrical and optical properties of the device and its underlying mechanism is investigated. Finally, the optimized parameters and conditions for device performance are concluded. 

## 2. Results and Discussion

In light emitting diodes, the injection and transport of electrons and holes and their recombination produce light emission. The structure of device used the model system in this study, comprising a known and efficient hole transport layer (HTL) and a usual doped polymer emissive layer (EML), is selected as a typical structure of a simple low cost solution processed PLED [8,27]. In this structure, holes are injected from the ITO electrode into the PEDOT-PSS buffer layer. By passing through it, they enter the PVK polymer layer. In addition, electrons are injected from the Al electrode to the PVK layer, and with the help of electrons transporting PBD, move through the polymer layer. In the final step, the recombination of electron-hole pairs in C6 dye emits light. 

The injection potential barrier plays an important role in determining the participation of charge carriers in the above-mentioned process. The barrier height for electron injection between Al and PVK layers is about 2 eV, while the barrier height for hole injection between ITO and PEDOT:PSS layers is about 0.4 eV [28]. So, electrons are the minority carriers in our current structure. 

### 2.1. Solvent Mixture 

**Figure 1:**
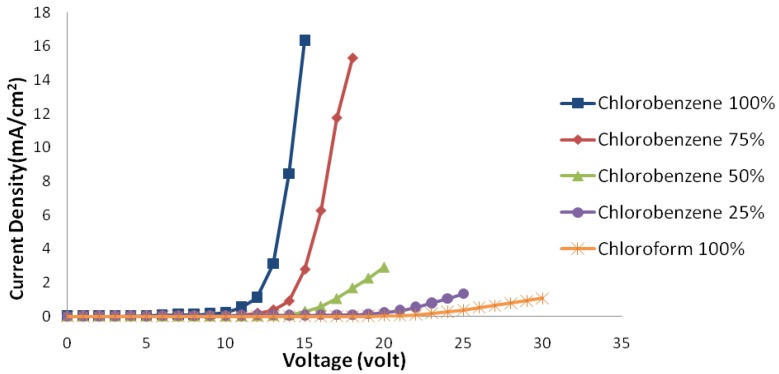
Electric current density-applied voltage curve of samples with different solvent mixture ratios in ambient conditions.

**Figure 2:**
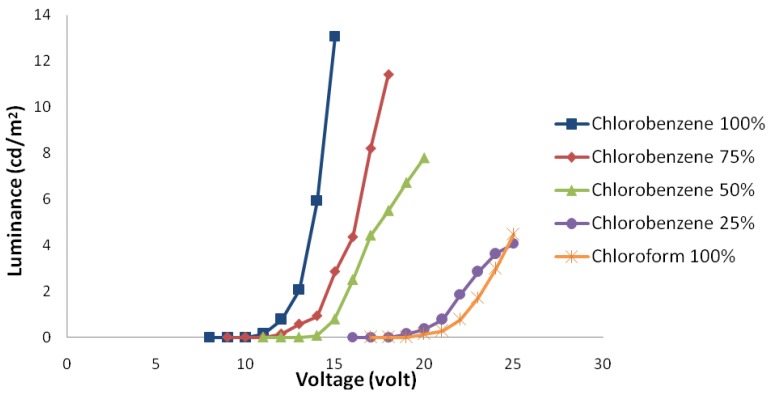
Luminance-applied voltage curve of samples with different solvent mixture ratios in ambient conditions.

Figure 1 shows electric current density-applied voltage characteristics and Figure 2 shows luminance-voltage characteristics of devices fabricated using chloroform-chlorobenzene solvent mixtures with different ratios. 

A sample spin coated from chloroform solvent has the highest threshold voltage. In addition, the amount of passing electric current and its luminance are low. By adding chlorobenzene, the threshold voltage decreases gradually, and at the same time, the amount of electric current and luminance increase. The main parameters effecting these changes are discussed and summarized below. 

#### 2.1.1. The Effect of Layer Thicknesses

The thickness of a spin coated layer depends on several parameters including the solvent evaporation rate. Vapor pressures of chloroform and chlorobenzene solvents at 21 °C are 169 and 9.5 mm Hg, respectively. Their vaporizing temperatures are 61 and 132 °C, respectively. In other words, at the same conditions, chloroform will be evaporated 5.25 times faster than chlorobenzene [29]. Since in spin coating, the evaporation rate has a direct relation with the layer thickness [11], adding chlorobenzene to chloroform decreases it. In practice, pure chlorobenzene layers are about 3 times thinner than pure chloroform ones. At a certain applied voltage, the amount of effective electric field depends on the layer thickness; thus, thinner layers feel higher effective electric fields. This, in its turn, leads to a decrease in the threshold voltage and an increase in the passing electric current of the device. 

#### 2.1.2. The Effect of Layer Surface Uniformities 

The solvent evaporation rate also highly influences surface uniformity. Low surface roughness and fluctuation using low evaporation rate solvents have been reported by other researchers [11]. In samples used in this study, the surface fluctuation of layers coated from pure chlorobenzene solvent is 4 times less than the surface fluctuation of samples coated from pure chloroform solvent. 

There may always be some current leakages in the system, but rough surfaces and defective ones are more likely to have leakages. Chloroform based systems are usually, as in this study, leakier and have more short circuited samples. 

Increasing surface uniformity of a polymer layer tends to its better contact with a metallic layer [16,30]. So, adding chlorobenzene to chloroform improves electron injection and hence increases luminance. In the current device structure, because of a lower potential barrier at the hole injection side, holes are the majority carriers. Electric current density-applied voltage characteristics depend mainly on majority carriers, but luminance-electric current density characteristics depend mainly on minority carriers [18]. 

Therefore, a lower solvent evaporation rate tends to form a thinner and more uniform polymer layer. In this situation, increased effective electric field and improved electron injection will increase the passing electric current and luminance and decrease the device threshold voltage. 

**Figure 3:**
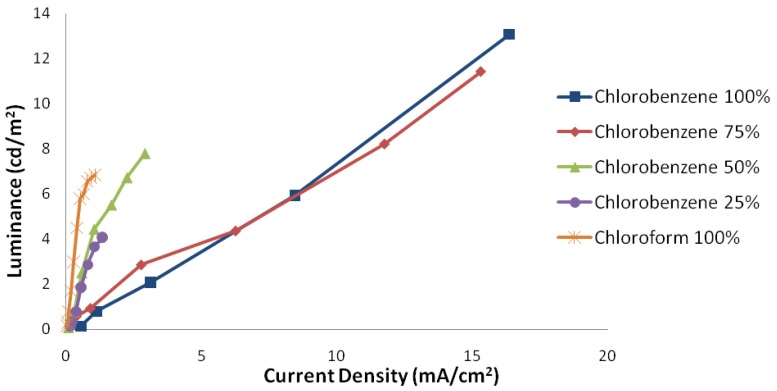
Luminance-electric current density curve of samples with different solvent mixture ratios in ambient conditions.

**Figure 4:**
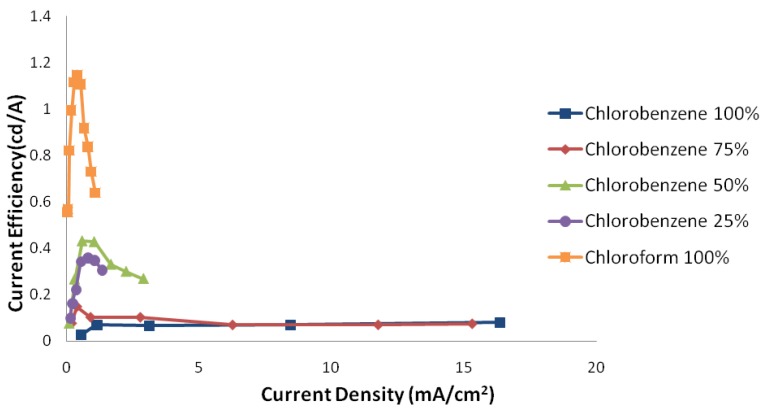
Electric current efficiency of luminescence-electric current density curve of samples with different solvent mixture ratios in ambient conditions.

Figure 3 and Figure 4 show the luminance-electric current density and electric current efficiency of luminescence-electric current density characteristics of devices, respectively. 

As luminance-electric current density characteristics show, although adding chlorobenzene increases the total carriers electric current, it does not increase luminance at the same rate. In other words, the increase in the amount of holes is more than electrons. The electric current efficiency of luminescence-electric current density characteristics implies this from another angle. 

The decrease of thickness tends to an increase in effective electric field, as mentioned earlier. The effect of this field is approximately the same for the both charge carriers. On the other hand, the improvement of the polymer-metal interface quality causes an increase in the electron injection. Considering only these two effects, the amount of electric current efficiency of luminescence in devices should be increased by adding chlorobenzene. 

To deal with this contradiction, another effective parameter is needed to be considered. Chloroform and chlorobenzene solvents, in addition to different evaporation rates, have different chemical structures; the chlorobenzene solvent, having benzene rings, is classified as an aromatic solvent, and the chloroform solvent is classified as a non-aromatic one. This difference has a great influence on the polymer chains arrangement and the nano-morphology of the final structure. 

#### 2.1.3. The Effect of Internal Nano-Structure Morphology 

In general, polymers consist of non-conductive single bound structures, conjugated structures and benzene rings. The solubility of each element depends on the interaction between solute and the solvent. Using the second rule of thermodynamics, the solubility of these two materials in each other could be as follows:

ΔG_M_= ΔH_M_ – TΔS_M_ < 0 (1)

where ΔG_M_, ΔS_M_ and ΔH_M_ are changes in the Gibbs free energy, entropy, and enthalpy of the system, respectively. T is absolute temperature of the system [17]. 

The amount of ΔS_M_ for solving two materials in each other is always positive. In the solving process, polymer solves in such a way that the system reaches its minimum free energy. So, to achieve better solubility, ΔH_M_ should be in its minimum amount. Changes in the internal energy of materials are the largest portion of enthalpy changes. 

When these two materials have similar chemical structures, ΔH_M_ reaches its minimum amount. Thus, mixing two aromatic or two non-aromatic materials causes minimal changes in enthalpy and they are more soluble in each other [17]. 

**Scheme 2:**
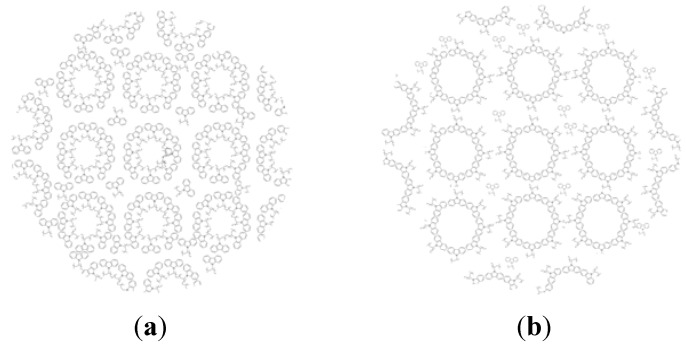
Schematic illustrations of internal nano-morphology for emitting layer of fabricated polymer light emitting diodes using an aromatic solvent (**a**) and a non-aromatic solvent (**b**).

According to this, the aromatic chlorobenzene solvent solves the aromatic part of PVK polymer better than its non-conductive main chain. Therefore, in different polymer chains, main chains are located close to each other and tend to stick one to another. This forms polymer regions with a non-conductive core of main chains and an aromatic shell of polymer branches. After spinning and the solvent evaporation, this morphology still survives [17,18]. In contrast, aromatic parts of different polymer chains get close together and approach each other in the non-aromatic chloroform solvent, constructing central cores of formed polymer regions. Non-conductive parts are located on the shells (Scheme 2). 

Therefore, using an aromatic solvent in the PLED structure tends to make contact between aromatic parts of polymer chain and electrodes. Due to conductivity of aromatic rings, potential barrier decreases and charge carrier injection improves. On the other hand, conductive parts of different polymer regions are located close to each other. Thus, charge carrier mobility of a host polymer, which is mainly hole transport in the current structure, increases. Considering that the distribution of PBD molecules is approximately unchanged, their overall function also does not change remarkably. In this way, the ratio of holes to electrons in the system increases. So, where electrons are minority carriers, using an aromatic solvent the electric current efficiency of luminescence decreases. 

### 2.2. Thermal Annealing Process 

The thermal treatment of thin layers could make nano-scale changes in the interfaces of layers and their internal nano-structure [16,17]. To prevent destructive side effects of long thermal treatment on organic materials, the annealing process should be performed in limited durations and temperatures. The glass transition temperature of undoped PVK is slightly above 200 °C; however, it is expected that the additives reduce it dramatically.

**Figure 5:**
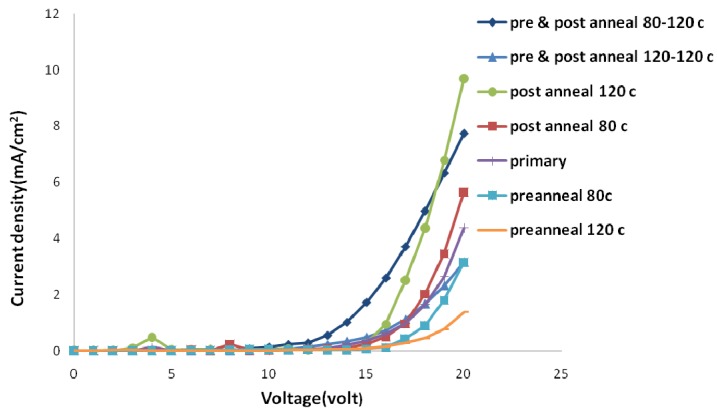
Electric current density-applied voltage curve of samples with different annealing temperatures in ambient conditions.

**Figure 6:**
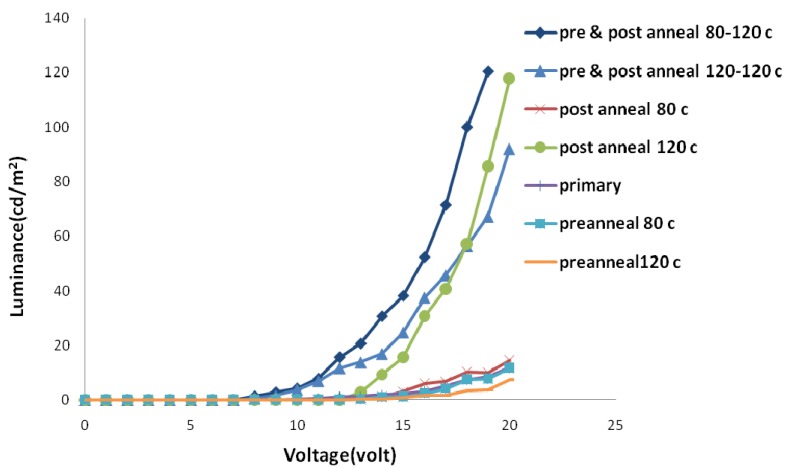
Luminance-applied voltage curve of samples with different annealing temperatures in ambient conditions.

The annealing process could be undertaken after spin coating and before the cathode deposition (pre-annealing) and/or after the metallization of a polymer surface (post-annealing). These two methods have different effects on sample properties. Figure 5 and Figure 6 show electric current density-applied voltage and luminance-applied voltage characteristics of devices fabricated using chloroform and chlorobenzene solvent mixtures containing the same weight ratio of its components (*i.e.*, 50/50). 

As Figure 5 and Figure 6 show, the pre-annealing process causes an increase in the threshold voltage and makes no noticeable change in the amount of luminescence. However, the threshold voltage decreases by post-annealing with the same trend when temperature increases from 80 to 120 °C. Moreover, a dramatic increase in luminance for samples post-annealed at 120 °C could be observed. Elevated temperatures destruct the aluminum surface and tend to produce dark spots on the emitting area of devices.

More investigations were performed through simultaneous pre- and post-annealing processes. We selected 80 °C and 120 °C for pre-annealing and 120 °C for post-annealing processes. The amount of electric current and luminance for samples pre-annealed at 80 °C was higher than those pre-annealed at 120 °C. Increasing pre-annealing temperature tends to degrade separate electrical and optical performances of devices. The surface roughness and fluctuation of samples pre-annealed at higher temperatures are more than other ones. 

The pre-annealing process tends to evaporate residual solvent from the polymer layer surface. Although this process enhances device stability, it increases the surface roughness and fluctuation of polymer layer. Thus, the quality of polymer-metal contact degrades. High surface roughness and fluctuation of polymer layer before cathode addition may cause some imperfections in deposition of a uniform and effective aluminum layer. 

Residual solvent will be evaporated in the post-annealing process too. However, this time, changes in the polymer layer surface are different due to the existence of metal cathode layer on the polymer layer. Thermal annealing causes the diffusion of metal layer into slightly melted polymer layer [31,32,33,34]. Whenever elevated temperatures approach glass transition temperature of polymer layer, the amount of this diffusion increases. Thereby, a graded interface is formed which comprises both a contact without imperfections and a geometrically non-planar electrode surface. 

At temperatures higher than 120 °C, residual solvent comes out of the polymer layer abruptly due to its increased evaporation rate, and damages the cathode surface seriously. So, 120 °C is set as the highest annealing temperature. 

It seems that nano-scale changes of the polymer-metal interface have the main role in improvement observed in the performance of fabricated organic light emitting devices. 

**Figure 7:**
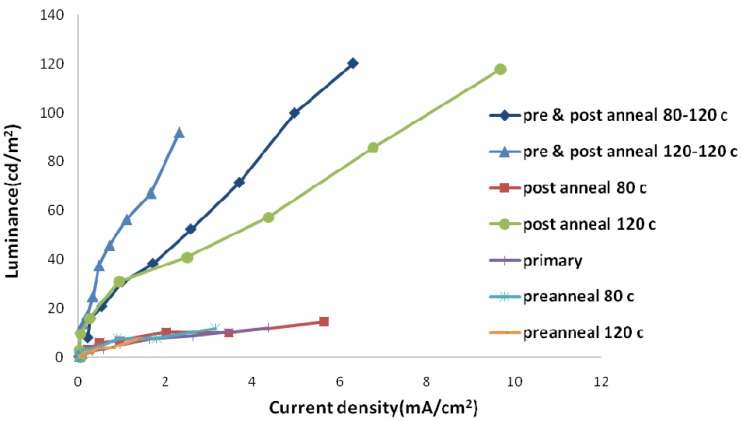
Luminance-electric current density curve of samples with different annealing temperatures in ambient conditions.

Figure 7 shows the amount of luminescence-electric current density at different annealing temperatures. Electric current efficiency of luminescence (the slope of the curve) is higher for three groups. In all of these three groups, post-annealing is performed at 120 °C. Increasing temperature up to 120 °C in the annealing process causes the effective diffusion of the metal layer into the polymer layer, and hence the improved electron injection into the polymer layer. As mentioned previously, electrons are minority carriers in the current OLED structure; thus, any improvement in their injection improves the device efficiency significantly. 

If the effective post-annealing process is accompanied with the pre-annealing process, residual solvent evaporation in the pre-annealing process prevents cathode surface damaging in the post-annealing process. Pre-annealing at higher temperatures tends to be more effective residual solvent removal. Therefore, the best electric current efficiency of luminescence is obtained in samples pre- and post-annealed at 120 °C. 

## 3. Experimental Section 

**Scheme 3:**
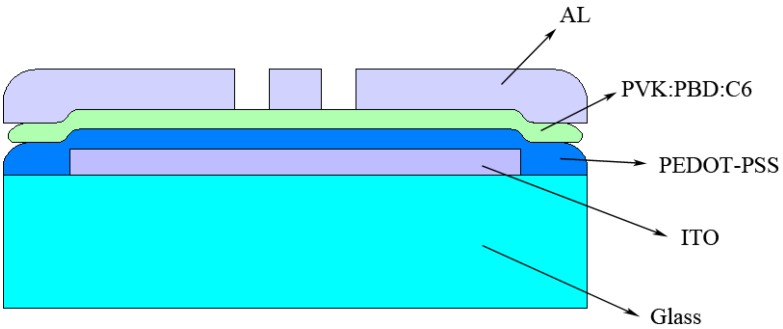
Schematic diagram of materials used for fabrication of polymer light emitting diodes by solution processing.

The structure of PLED is Glass/ITO/PEDOT-PSS/PVK:PBD:C6/Al (Aldrich). First, indium tin oxide (ITO) coated glass is washed in an ultrasonic bath by pure water, acetone and propanol, respectively. Then, the aqueous solution of poly-(styrene sulfonate) doped poly-(3,4-ethylene dioxythiophene) (PEDOT-PSS) polymer is spun onto it to form a 40 nm thick layer. High molecular weight poly-(9-vinyl carbazole) (PVK) polymer, organic small molecule 2-(4-biphenyl)-5-(4-t-butylphenyl)-1,3,4-oxadiazole (PBD) and 3-(2-Benzothiazolyl)-N,N-diethylumbelliferylamine, 3-(2-Benzothiazolyl)-7-(diethylamino)-coumarin (Coumarin 6 or C6) dye (100:40:0.03 weight ratio) are solved in the chloroform-chlorobenzene solvent mixture, and are spun (in less than 100 to more than 200 nm thickness) onto the PEDOT-PSS layer. At last, the Aluminum (Al) metal layer is deposited in 150 nm thickness on the polymeric bilayer by evaporation (Scheme 3).

Annealing of samples at different temperatures is performed in an oven with the temperature controlling ability. Three procedures for thermal annealing are used; pre-annealing, post-annealing and both pre- and post-annealing. In the pre-annealing process, samples are annealed at 80 °C and 120 °C for 5 min before metallization. Samples are annealed at 120 °C for 5 min after Al deposition in post-annealing process. The thicknesses of the layers and their surface roughnesses are measured using conventional methods with a stylus surface profile meter (Dektak) and an atomic force microscope (AFM). Electrical measurements are carried out by the Keithley 6487 picoammeter/voltage source unit, and optical measurements are taken by a photometer, a spectrometer, and a standard setup which consists of a photodiode assembled with an amplifier connected to an oscilloscope. All the fabrication and characterization processes are conducted under laboratory ambient conditions. 

## 4. Conclusions 

The type of solvent and its properties affect electrical and optical properties of solution processed PLEDs. Mixing solvents could be an effective way for tuning these properties. Adding aromatic chlorobenzene solvent with a low evaporation rate to non-aromatic chloroform solvent with a high evaporation rate makes nano-scale polymer layer thinner and more uniform. This, in turn, increases effective electric field and improves electron injection. In this way, it increases the passing electric current and luminance and decreases the threshold voltage of devices. Moreover, it makes a contact between aromatic parts of polymer chains and electrodes, so charge injection potential barrier decreases. Conductive parts of formed polymer regions are located close to each other; thus, charge carrier mobility in a PVK polymer host which is mainly hole transport improves. Thereby, the ratio of holes to electrons in the system increases. Also, in the current device structure, holes are the majority carriers due to their lower injection potential barrier; so electric current efficiency of luminescence decreases. 

In the thermal annealing process, residual solvent evaporation makes some nano-scale changes in layer interface. Pre-annealing increases the surface roughness and fluctuation of the polymer layer and degrades electron injection. High surface roughness and fluctuation of the polymer layer make some difficulties in effective metal deposition. Post-annealing causes the diffusion of aluminum layer into the polymer layer, and thus improves electron injection. Using simultaneous pre- and post-annealing processes leads to increase electric current efficiency of luminescence. In this case, pre-annealing at higher temperatures tends to be more effective residual solvent removal, and the post-annealing process at higher temperatures causes an effective diffusion of the metal layer into the polymer layer. Therefore, the best electric current efficiency of luminescence is obtained in samples pre- and post-annealed at 120 °C. In these samples, the amount of luminance increase to the electric current change ratio is more than other fabricated devices.
